# Molecular mechanisms of activating c-MET in KSHV+ primary effusion lymphoma

**DOI:** 10.18632/oncotarget.15444

**Published:** 2017-02-17

**Authors:** Bao Quoc Lam, Lu Dai, Li Li, Jing Qiao, Zhen Lin, Zhiqiang Qin

**Affiliations:** ^1^ Department of Genetics, Louisiana State University Health Sciences Center, New Orleans, LA 70112, USA; ^2^ Louisiana Cancer Research Center, Louisiana State University Health Sciences Center, New Orleans, LA 70112, USA; ^3^ Department of Pediatrics, Research Center for Translational Medicine and Key Laboratory of Arrhythmias, East Hospital, Tongji University School of Medicine, Shanghai 200120, China; ^4^ Department of Pathology, Tulane University Health Sciences Center, Tulane Cancer Center, New Orleans, LA 70112, USA

**Keywords:** KSHV, primary effusion lymphoma, c-MET, Plexin-B1, SNV

## Abstract

The oncogenic Kaposi's sarcoma–associated herpesvirus (KSHV) is a principal causative agent of primary effusion lymphoma (PEL), which is mostly seen in immunosuppressed patients. PEL is a rapidly progressing malignancy with a median survival time of approximately 6 months even under the conventional chemotherapy. We recently report that the hepatocyte growth factor (HGF)/c-MET pathway is highly activated in PEL cells and represents a promising therapeutic target (*Blood. 2015;126(26):2821-31*). However, the underlying mechanisms of c-MET activation within PEL cells remain largely unknown. To solve this puzzle, here we have utilized the next generation sequencing (NGS) based bioinformatics approach to investigate the genomic landscape of the c-MET gene and we found that there's no single nucleotide variations (SNVs) occurred in the c-MET genomic regions in a cohort of PEL samples. Consistently, Sanger sequencing analysis of frequently mutated exons such as exon 10, 14 and 19 shows no mutation of these c-MET exons in PEL cell-lines, which further supports the notion that mutations are not the major mechanism responsible for c-MET activation in PEL. Further, we found that a transmembrane receptor protein, Plexin-B1, is expressed in PEL cell-lines, which is required for c-MET-mediated PEL cell survival via direct protein interaction.

## INTRODUCTION

The DNA tumor virus, Kaposi's sarcoma-associated herpesvirus (KSHV) can cause several human cancers especially in the immunocompromised populations [1]. One such malignancy is primary effusion lymphoma (PEL) which is derived from the KSHV-transformed B cells, in the pleural or peritoneal cavities of HIV+ patients [2]. To date, the prognosis of PEL is very poor, and the patients’ median survival time is only about couples of months even following active therapeutic interventions [3]. Currently, the combination of cytotoxic chemotherapies is the routine regimen for PEL management, but the high toxicity caused by systemic chemo, antiretroviral and/or immune suppression therapies, greatly decreases the benefits of these treatments [4, 5].

One of receptor tyrosine kinases (RTKs), c-MET, is encoded by the *Met* proto-oncogene, and the hepatocyte growth factor (HGF) is the only known c-MET ligand [6, 7]. Since the discovery of c-MET in the 1980s, the c-MET pathway has gained considerable interest related to a variety of cancers due to the diversity of the cellular responses that follow c-MET activation, including cell growth, survival, motility, invasion, and metastasis [8]. Multiple mechanisms turn out to be responsible for the aberrant activation of c-MET, such as overexpression of c-MET gene, increased c-MET gene copy number, mutations which constantly activate c-MET, stimulation caused by an increased autocrine or paracrine, as well as the interaction between c-MET and other receptors on the cell surface [9]. Previous literatures have reported that missense mutations and SNVs are mainly located in the SEMA and juxtamembrane domain of c-MET, on the other hand, activating mutations have been identified in limited types of solid tumors, such as hereditary and spontaneous renal carcinomas, non–small cell lung cancers (NSCLC), hepatocellular carcinomas, etc [10–15]. Candidate tumorigenic mutations include: 1) point mutations that produce a c-MET splicing variant missing the exon 14 encoded-juxtamembrane domain [13, 16]; 2) point mutations in the c-MET kinase domain resulting in the constitutive activation [12]; 3) Y1003 mutations which inactivate the Cbl binding site and causes uncontrolled c-MET expression [17].

We recently showed an abnormal activation of the HGF/c-MET signaling pathway within KSHV+ PEL cell-lines [18]. One of the selective c-MET inhibitors, namely PF-2341066, can significantly induce PEL cell apoptosis by inducing cell cycle arrest and DNA damage response, and can effectively inhibit PEL progression in a xenograft immunodeficiency murine model [18]. Recently, some strategies targeting c-MET kinase activity have been developed and tested in multiple clinical trials, including small-molecule inhibitors and monoclonal antibodies, for a variety of solid tumors [8, 9]. However, the underlying molecular mechanisms for c-MET activation remain largely unknown in these special virus-associated lymphoma cells. Here, we set to investigate the mutation landscape of c-MET gene within KSHV+ PEL samples, using the NGS based bioinformatics approaches. In parallel, we also tried to understand the role of other cellular receptors in the activation of c-MET from PEL cells, especially Plexin-B1.

## RESULTS AND DISCUSSION

### NGS based SNVs analysis of c-MET in PEL samples

**Figure 1:**
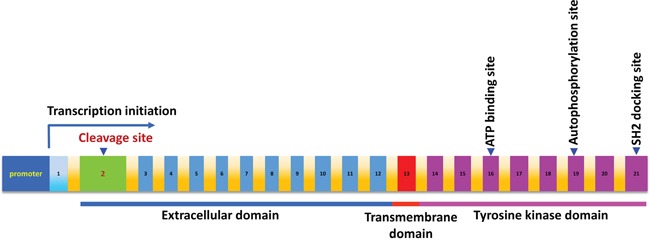
The schematic diagram of MET proto-oncogene The position of the cDNA sequence is defined according to GenBank #J02958, which contains 21 exons shown by the numbers. The position of the ATP binding site, autophosphorylation site and SH2 docking sites are indicated by an arrow, respectively.

**Figure 2:**
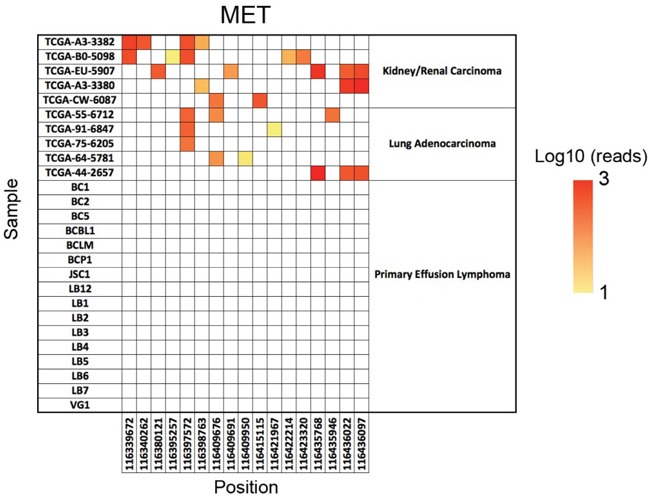
The SNV analysis of c-MET The positions of all single nucleotide variations (SNVs) across the c-MET genomic regions are on the *x* axis and the sample name on the *y* axis. Dots indicate a nucleotide difference from the hg19 reference genome, and the color of the dots indicates the number of reads covering that specific SNV on a log10 scale. The TCGA kidney/renal carcinoma and lung adenocarcinoma data sets were used as the positive controls.

The human c-MET gene consists of 21 exons and encodes a protein of 1408 amino acids (aa). Structurally, the c-MET protein contains both extracellular and intracellular domains. Its extracellular domain can be further divided into the semaphoring domain, the plexin-semaphorin-integrin domain, and four immunoglobulin-plexin-transcription domains. The intracellular domain of c-MET carries its tyrosine kinase activity (Figure 1) [6, 19]. To date, it is still unclear whether the c-MET gene harbors any oncogenic SNVs and thus leads to its abnormal activation in PEL cells. To investigate the genomic landscape of the c-MET gene, here we have analyzed a cohort of PEL data sets (16 cell-lines isolated and established from HIV+ or HIV- PEL patients, accession no. SRP032975) using a well-established NGS approach as described in the Methods. Our results show that there are no single nucleotide variations (SNVs) in the coding sequence (cds) regions of c-MET, whereas c-MET SNVs were readily detected in the control TCGA kidney/renal carcinoma and lung adenocarcinoma samples (Figure 2). Nevertheless, we indeed noticed that the effective sequencing depth of the c-MET gene of these PEL data sets is significantly lower (around 142-fold lower) than those control TCGA data sets. Thus, it is possible that some potential c-MET mutations may be detectable by using some high-quality deep-sequencing datasets in the future. However, due to the rarity of this disease, these are the only published PEL NGS data sets we can acquire for analysis.

### Sanger sequencing of representative exons of c-MET in PEL cell-lines

**Figure 3:**
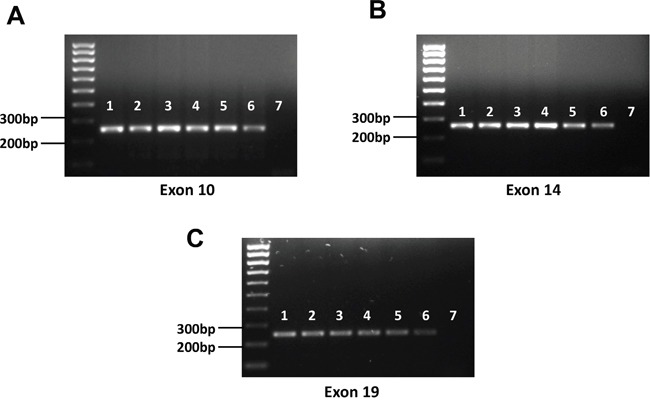
Detection of representative exons of c-MET from PEL cell-lines **A-C**. Genomic DNA was extracted from PEL and NSCLC cell-lines as described in the Methods and PCR was used to detect representative exons (10, 14 and 19) of c-MET. 1: BC-1; 2: BC-3; 3: BCP-1; 4. BCBL-1; 5: JSC-1; 6: A549 (positive control); 7: negative control.

**Figure 4:**
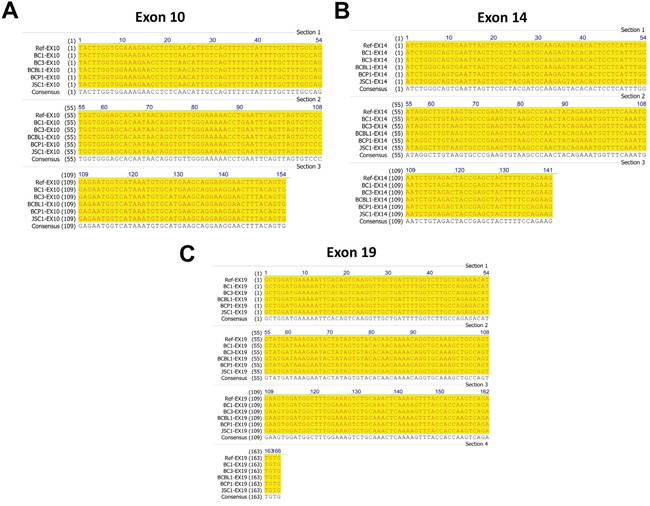
Sequencing and alignment analysis of representative exons of c-MET from PEL cell-lines **A-C**. PCR products of exon 10, 14 and 19 of c-MET amplified from five PEL cell-lines were purified and sequenced as described in the Methods. The alignment analysis was performed using Vector NTI v10.0 software (Invitrogen), and the human c-MET sequence (GenBank #J02958) used as the reference sequence.

To further confirm our SNVs analysis results, we performed Sanger sequencing of exon 10, 14 and 19 of c-MET, all of which have been reported frequent mutations in solid tumors as mentioned above. Our PCR results showed all the 5 examined KSHV+ PEL cell lines (BC-1, BC-3, BCP-1, BCBL-1 and JSC-1) having the same length of exon 10, 14 and 19 as that from A549, one of NSCLC cell-lines without c-MET missense mutations [20] (Figure 3). The Sanger sequencing of these c-MET exons PCR products and alignment analysis confirmed no detectable mutations within all the 5 PEL cell-lines, when compared to the human c-MET reference sequence (GenBank #J02958) (Figure 4). Taken together, our data indicate that mutations are not the major molecular mechanism responsible for c-MET activation in PEL tumor cells.

### The expression of Plexin-B1 and its interaction with c-MET within PEL cell-lines

**Figure 5:**
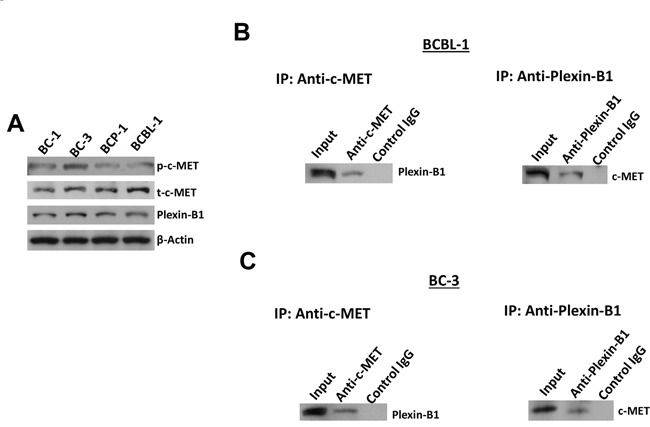
The interaction of c-MET and Plexin-B1 proteins within PEL cell-lines **A**. The protein expression within 4 PEL cell-lines was measured by using immunoblots. **B-C**. Bi-directional immunoprecipitation was used to detect the interaction of c-MET and Plexin-B1 proteins within BCBL-1 and BC-3 cell-lines as described in the Methods.

As mentioned above, c-MET can be activated through interaction with other active cell-surface receptors. Previous studies have shown that the Plexin B family proteins such as Plexin-B1 are able to bind to the HGF RTK proteins, c-MET and RON [21, 22]. These Plexin B proteins are transmembrane receptors with single pass and they share a good structural similarity with c-MET [23, 24]. Furthermore, Plexin-B1 can be activated by its high-affinity ligand Sema4D, and such activation can subsequently transactivate c-MET and promote tumor growth, invasion, migration and angiogenesis [21, 22, 25]. Therefore, we first examined the expression of Plexin-B1 in 4 KSHV+ PEL cell-lines, namely BC-1, BC-3, BCBL-1 and BCP-1. And our results showed that expression of Plexin-B1 was detected in all the examined cell-lines, although Plexin-B1 also shows a differential expression among these cells (Figure 5A). Next, by using bi-directional immunoprecipitation assays, we found the interaction of Plexin-B1 and c-MET proteins within BCBL-1 and BC-3 cell-lines (Figure 5B-5C).

### Plexin-B1 is required for c-MET phosphorylation and related cellular functions within PEL cells

**Figure 6:**
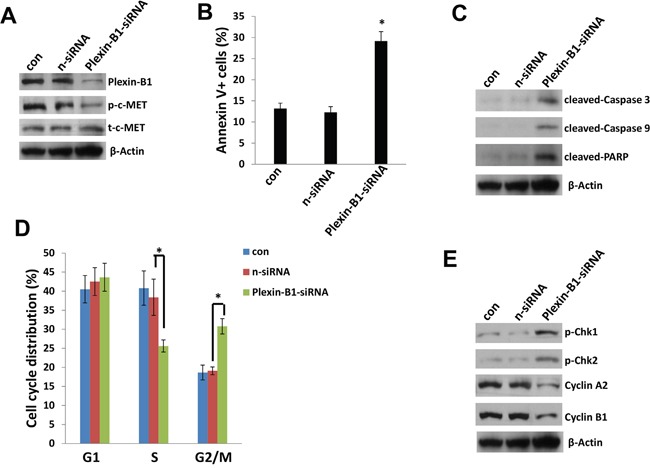
Plexin-B1 is required for c-MET activation and mediated functions within PEL cells **A, C, E**. BC-3 cells were first transfected with either negative control siRNA (n-siRNA) or *Plexin-B1*-siRNA for 48 h, then protein expression was measured by using immunoblots. **B, D**. Cell apoptosis and cell cycle were measured by using flow cytometry as described in the Methods. Error bars represent the S.E.M. for 3 independent experiments. * = p<0.05.

To further understand the role of Plexin-B1 in c-MET mediated functions in PEL cells, we successfully “knocked-down” Plexin-B1 expression using smart-pool siRNAs in BC-3 cell-line, which having the highest level of c-MET phosphorylation in the 4 PEL cell-lines we tested (Figure 5A and Figure 6A). We found that silencing of Plexin-B1 dramatically reduced the c-MET phosphorylation but not total c-MET expressional level within BC-3 cells (Figure 6A). We recently reported that one of selective c-MET inhibitors, PF-2341066 treatment caused PEL cell cycle arrest and apoptosis [18]. Here we found that silencing of Plexin-B1 also induced BC-3 cell apoptosis which is caspases/PARP-dependent (Figure 6B-6C). Cell cycle analysis indicated that silencing of Plexin-B1 significantly increased G2 phase subpopulation while reducing S phase subpopulation of BC-3 cells. Immunoblots results confirmed that silencing of Plexin-B1 increased the expression of check-point regulatory proteins such as p-Chk1 and p-Chk2, while reducing Cyclin A2 and Cyclin B1 expression from BC-3 cells (Figure 6D-6E). Taken together, our data first time demonstrate that Plexin-B1 is required for c-MET activation and related cellular functions for PEL survival, which may represent a promising anti-cancer target. Nevertheless, we think that additional cellular factors are potentially involved in the activation of c-MET in PEL cells. For instance, Orian-Rousseau *et al* have reported that CD44v6, a CD44 splicing isoform carries variant exon v6 sequences, and it plays a critical role in the activation of c-MET in both primary keratinocytes and carcinoma cells of rat and human [26]. Interestingly, our previous study has found that there is very low level of pan-CD44 expression within PEL cell-lines [27]. However, the presence of CD44v6 and its regulatory role in c-MET activation for PEL cells still require further investigation.

## MATERIALS AND METHODS

### Cell culture and reagents

The PEL cell-line BCBL-1 (KSHV+/EBV-) was cultured as described previously [18]. The other PEL cell lines BC-1 (KSHV+/EBV+), BC-3 (KSHV+/EBV-), BCP-1 (KSHV+/EBV-), JSC-1 (KSHV+/EBV+) were purchased from American Type Culture Collection (ATCC), and maintained in complete RPMI 1640 medium (ATCC) supplemented with 20% FBS. The NSCLC cell-line, A549 was purchased from ATCC and cultured in RPMI-1640 medium (Gibco) supplemented with 10% fetal bovine serum and 1% penicillin & streptomycin [28]. All cells were incubated at 37°C in 5% CO_2_. All experiments were carried out using cells harvested at low (<20) passages.

### SNV analysis

Raw whole exome sequencing reads from five kidney/renal carcinoma samples (Patient ID# TCGA-A3-3382, TCGA-B0-5098, TCGA-EU-5907, TCGA-A3-3380 and TCGA-CW-6087) and five lung adenocarcinoma samples (Patient ID# TCGA-55-6712, TCGA-91-6847, TCGA-75-6205, TCGA-64-5781 and TCGA-44-2657) generated through the National Institutes of Health, The Cancer Genome Atlas (TCGA) project were obtained from the National Cancer Institute Genomic Data Commons. Raw sequencing reads from 16 PEL cell-lines were obtained from the National Center for Biotechnology Information short reads archive (accession no. SRP032975). Raw sequence data were first aligned to the human reference genome hg19 with the Burrows-Wheeler Aligner. Next, the resulting alignment files were analyzed using a detection pipeline consisting of Samtools mpileup and VarScan to identify candidate SNVs. Lastly, the identified SNVs were annotated using the ANNOVAR software and the c-MET specific SNVs were then plotted as a heatmap based on the number of reads covering that specific SNV on a log10 scale.

### DNA extraction, PCR and sequencing

Genomic DNA from PEL cells was extracted using the Wizard Genomic DNA Purification Kit (Promega) according to the manufacturer's instructions. Primers used for amplification of specific exons of c-MET gene are listed in Table 1. Amplification experiments were carried out on a Biometra T3000 Thermocycler, under conditions of 94°C for 5 min, 35 cycles of 94°C for 30 s, 54°C for 30 s, and 72°C for 60 s. Amplicons were subsequently identified by ethidium bromide-loaded agarose gel electrophoresis. PCR productions were purified by using QIAquick PCR Purification Kit (QIAGEN), prior to being sequenced on an Applied Biosystems™ 3130xl Genetic Analyzer. Sequencing data analysis was performed using Sequencing Analysis Software v6.0 (Applied Biosystems). Sequence alignment analysis was performed using Vector NTI v10.0 software (Invitrogen).

**Table 1:** Primer sequences for PCR in the current study

Gene	Sequences	Sequences (5’ → 3’)
*MET Exon 10*	*sense*	*ctctgacctgtaatcagtgca*
	*antisense*	*gttagaggcaaagatgcagag*
*MET Exon 14*	*sense*	*gggcccatgatagccgtctttaa*
	*antisense*	*tacacaacaatgtcacaaccca*
*MET Exon 19*	*sense*	*ttctatttcagccacgggtaat*
	*antisense*	*atgaaagtaaaagaggagaaactc*

### RNA interference

For RNA interference, *Plexin-B1* ON-TARGET plus SMART pool siRNA, or negative control siRNA (n-siRNA) (Dharmacon), were delivered using the DharmaFECT transfection reagent according to the manufacturer's instructions.

### Flow cytometry

For cell apoptosis assays, the fluorescein isothiocyanate (FITC)–Annexin V and propidium iodide (PI) Apoptosis Detection kit I (BD Pharmingen) was used as previously described [29]. For cell cycle analysis, PEL cell pellets were fixed in 70% ethanol, and incubated at 4°C overnight. Cell pellets were re-suspended in 0.5 mL of 0.05 mg/mL PI plus 0.2 mg/mL RNaseA and incubated at 37°C for 30 min. Cell cycle distribution was analyzed on a FACS Calibur 4-color flow cytometer (BD Bioscience).

### Immunoblotting and immunoprecipitation

Total cell lysates (20μg) were resolved by 10% SDS–PAGE, transferred to nitrocellulose membranes, and immunoblotted with antibodies for cleaved caspase 3/9, cleaved PARP, phosphor (p)-c-MET/total (t)-c-MET, p-Chk1/2, Cyclin A2, Cyclin B1 (Cell Signaling), Plexin-B1 (Santa Cruz), and β-Actin (Sigma) for loading controls. Immunoreactive bands were identified using an enhanced chemiluminescence reaction (Perkin-Elmer), and visualized by autoradiography. Immunoprecipitation assays were performed using Catch and Release Immunoprecipitation Kit (Milipore) according to the manufacturer's instructions.

### Statistical analysis

Significance for differences between experimental and control groups was determined using the two-tailed Student's t-test (Excel 8.0), and p values <0.05 were considered significant.
